# Pilot Study: Increasing Confidence in Obstetrics and Gynecology Applicants Through a Prep Course Prior to Audition Rotations

**DOI:** 10.7759/cureus.27793

**Published:** 2022-08-08

**Authors:** Danielle Wright, Jessica Bailer, Amanda Hall, Halle Lindsey, Brelahn Wyatt

**Affiliations:** 1 Obstetrics and Gynecology, Uniformed Services University of the Health Sciences, Bethesda, USA

**Keywords:** step 1 pass/fail, graduate medical education (gme), skills interns, gynecology and obstetrics, teaching bootcamp, medical school education

## Abstract

Introduction: As more medical schools schedule the United States Medical Licensing Examination (USMLE) Step 1 after the completion of clinical rotations in their curriculum, students are finding themselves increasingly removed from the clinical setting prior to their audition rotations. In the military, these audition rotations are important for matching into competitive specialties, such as Obstetrics and Gynecology (Ob-Gyn). This pilot study explores the confidence and audition readiness of prospective candidates through their participation in an Ob-Gyn preparatory course.

Methods: Rising fourth year medical students applying for an Ob-Gyn residency attended an in-person review session. It consisted of four interactive presentations addressing rotation resources, labor and delivery triage, postpartum care, laparoscopic anatomy review, and concluded with a resident question and answer panel. These attendees completed a pre- and post-course survey on Google Forms. Categorical answers were recorded as response frequency and Likert scales were converted into a 5-point system for analysis.

Results: A 100% response rate from attendees revealed pre-course 81.8% confidence in performing well on an audition rotation with only 27% feeling prepared in terms of their medical knowledge. After completion of the course, all participants reported increased medical knowledge and recommended the course to other students.

Conclusions: Preparatory courses for Ob-Gyn residency candidates can increase confidence and preparedness for audition rotations and, ultimately, internship. As the residency application process becomes more competitive, departments can take steps such as hosting a preparatory course to best assist their students into matching and provide skills that they can practice heading into internship.

## Introduction

In the United States (US) and Canada there are 171 accredited medical schools [1]. Their curricula are often changing in structure and content, which is influenced by accreditation, the United States Medical Licensing Examination (USMLE), competency frameworks for assessment, and strategies for individual feedback [2,3]. Notable trends in curriculum changes include an emphasis on early patient contact and with this goal medical educators are faced with the challenge of determining the optimal timing of USMLE Step 1 [2].

A growing number of medical schools are placing Step 1 after core clinical rotations instead of after the completion of the first two preclinical years due to the greater degree of clinical content on the test in addition to its standard basic science content [4]. Outcomes of this change include a small increase in Step 1 scores and a reduction in failure rates [4,5]. Previously, Step 1 scores translated into residency opportunities; however, with the National Board of Medical Examiners’ decision to change Step 1 to Pass/Fail, it is unclear if changing the timing of Step 1 will have a meaningful impact [6-9]. At this time, there is limited research that explores how the timing of Step 1 affects student confidence and performance on advanced clinical rotations and audition interviews as the prolonged time away from the hospital floor to study could decrease their sense of readiness. Hence, student confidence during this timeframe should be explored. 

In the United States military, the match process varies from the civilian match because of its emphasis on audition rotations which allow students to showcase their clinical skills as well as their function within a team and receptiveness to feedback. Because audition rotations influence selection into a residency program it is reasonable to expect applicants desire to feel prepared. Given the timing of Step 1, which can prolong time away from clinical medicine, students could sense a decrease in their clinical readiness. This pilot study aims to showcase our curriculum that prepares and re-engages students in Obstetric and Gynecologic (Ob-Gyn) content prior to audition rotations serving to boost their confidence and help them match.

## Materials and methods

Study design

This study was reviewed by the Institutional Review Board (IRB) of the Uniformed Services University of the Health Sciences (USUHS) and Walter Reed National Military Medical Center and it was considered to be exempt (approval number: DBS.2022.351). In February 2022, all fourth-year medical students interested in applying to Ob-Gyn at USUHS (N=11) were connected with a faculty mentor who was encouraged to do a resume review and mock interview with each mentee. In March 2022, all 11 students attended a 2.5-hour in-person session comprising five interactive presentations covering: 1) rotation resources, 2) labor and delivery triage, 3) postpartum care, 4) laparoscopic anatomy review, and 5) a resident question and answer panel. The lectures on resources, triage, and postpartum care were delivered through PowerPoint presentations (Microsoft, Redmond, WA, USA); the anatomy review was done through Kahoot (Kahoot!, Oslo, Norway). The students did not get feedback on their performance during this session.

Consent

Students were asked to complete an optional pre- and post-course survey using Google Forms. Written informed consent was waived by the IRB, as the survey was considered to be program evaluation used to determine the course's utility in preparing students for their audition rotations. Oral consent to partake in the survey was obtained by each participant and documentation of consent was made by way of their participation in the survey.

Analysis

Questions were posed to students using a scale from “Strongly Disagree” to “Strongly Agree.” These results were then converted to a 5-point scale for analysis with higher scores reflecting a consensus of "Agree" or "Strongly Agree." The average response along with the standard deviation is reported. Categorical data were reported by response frequency (%). 

## Results

**Figure 1:**
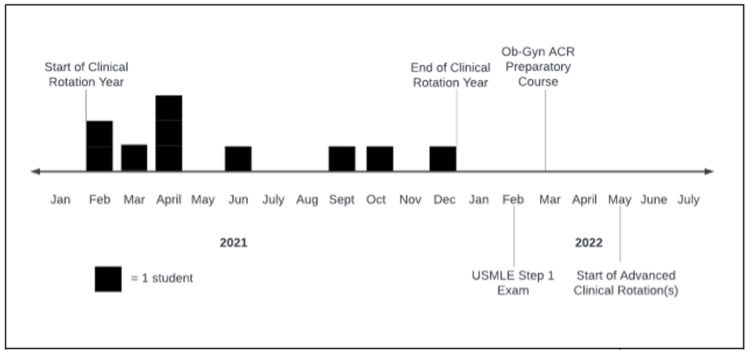
Medical Student Clinical Timeline Timeline depicting the time between each student's core Ob-Gyn clerkship, Step 1, and their first interview rotation.

Eleven students participated in the course; we had a 100% response rate. On average, there were 11.4 ± 3.5 months between their Ob-Gyn core clinical rotation (i.e. third year) and their first Ob-Gyn advanced clinical (i.e. fourth year) rotation (Figure 1). Before the preparatory course, 81.8% of students reported feeling confident that they will perform well on their Ob-Gyn rotations though 27% of students reported feeling prepared in terms of their medical knowledge (Table 1). After the course, 100% of students reported increased preparedness in terms of their medical knowledge and reported the course met their expectations (Table 2). Based on the average ratings from the post-course survey, the majority of students felt the course was well organized and helpful towards them achieving their goal of matching; the average rating for perceived increased confidence in performing well on audition rotations was 4.36 ± 0.50 (Table 3).

**Table 1:** Pre-course Survey Responses Pre-course responses from the preparatory session reported as frequency (%).

In terms of my medical knowledge, I feel prepared for my advanced clinical rotation(s) in Gynecologic Surgery and Obstetrics.	Students (%)
No, I do not feel prepared	3 (27)
Neutral, I’m indifferent	5 (45)
Yes, I feel prepared	3 (27)
I am confident that I will perform well on my advanced clinical rotation{s) in Gynecologic Surgery and Obstetrics.	
No, I do not feel confident that I will perform well	0 (0)
Neutral, I'm indifferent	2 (18)
Yes, I feel confident that I will perform well	9 (82)

**Table 2:** Post-course Survey Responses Post-course responses from the preparatory session reported as frequency (%).

In terms of my medical knowledge, I feel more prepared for my advanced clinical rotation(s) in Gynecologic Surgery and Obstetrics.	Students (%)
No, I do not feel more prepared for my future rotations	0 (0)
Yes, I feel more prepared for my future rotations	11 (100)
Overall this course met my expectations.	
Yes, this course met my expectations	11 (100)
No, this course did not meet my expectations	0 (0)

**Table 3:** Post-course Likert Scale Responses Post-course Likert scale responses were originally recorded as follows:  1- Strongly disagree, 2 - Disagree, 3 - Neutral, 4 - Agree, 5 -Strongly agree.  These responses were converted to a 5-Point Scale; the average response is reported along with the standard deviation (STD).

	Average Rating (STD)
The course was organized in a manner that helped me understand the underlying concepts	4.72 (0.46)
The course has increased my confidence in performing well on my advanced clerkship rotation(s) in Gynecologic Surgery and Obstetrics	4.36 (0.50)
This course was helpful towards my progress in matching into Gynecologic Surgery and Obstetrics	4.36 (0.50)
I would highly recommend this course to other students	4.90 (0.30)

## Discussion

In 2021, a total of 2,756 applications (out of 50,830 across all specialties) were submitted for residency in Ob-Gyn for 1,460 positions offered in 279 programs. According to the Association of American Medical Colleges, Ob-Gyn ranked in the top five specialties for both US and international medical graduates [10]. With increasing interest in Ob-Gyn, emphasis on successful applications and interviews is imperative to matching.

In the US military, the match process varies from the civilian match because of its emphasis on audition rotations that allow students to showcase their clinical skills, work ethic, and overall function within a team [11,12]. Because audition rotations influence selection into a program it is reasonable to expect applicants to desire to feel prepared; from the data our students did own a sense of doubt in regards to their medical knowledge in Ob-Gyn content prior to the start of their advanced clinical rotations despite having taken Step 1 (and sometimes Step 2 as well). Lerner and colleagues (2018) highlighted that a surgical simulation-based elective course was an effective tool for helping third- and fourth-year medical students transition to Ob-Gyn residency; additionally end-of-fourth-year bootcamps have been evaluated in other specialties and noted to improve student confidence, clinical knowledge, and procedural skill level prior to the start of residency [13-15]. 

This pilot study surveyed prospective applicants’ confidence before and after a preparatory course that aimed to not only mentor students but also to reengage them in Ob-Gyn content prior to their fourth-year rotations and we achieved our goal for them to have a sense of increased preparedness. This curriculum advocates for a "pre-audition rotation bootcamp” in addition to the traditional "pre-internship bootcamp" that is usually held in the summer prior to the start of internship. Benefits of an early-fourth-year bootcamp include: incorporating a formal mentorship program that includes mock interviews/resume review allowing a leveling of the playing field in terms of interview preparation, highlighting specific skills early allows them to practice throughout their fourth year ultimately improving their performance during internship, and this course could close the equity gap for medical students from traditionally marginalized communities who may experience anxiety when seeking help in preparation for audition rotations, interviews, and internship. Limitations of this study include the small sample size and lack of long-term follow-up from participants to include their success in matching.

## Conclusions

Few studies have assessed the impact early-fourth-year bootcamps can have on match success. Based on the results of this study, this type of preparation can greatly improve the confidence and preparedness of Ob-Gyn applicants. As matching into any specialty increasingly becomes more competitive, medical schools should continue to assess how they can make their fourth years more competitive for their residency of choice.
